# Recent Advances in Fluorescence Recovery after Photobleaching for Decoupling Transport and Kinetics of Biomacromolecules in Cellular Physiology

**DOI:** 10.3390/polym14091913

**Published:** 2022-05-07

**Authors:** Ning Cai, Alvin Chi-Keung Lai, Kin Liao, Peter R. Corridon, David J. Graves, Vincent Chan

**Affiliations:** 1 Wuhan Institute of Technology, School of Chemical Engineering and Pharmacy, Wuhan 430073, China; cain0001@e.ntu.edu.sg; 2 Department of Architecture and Civil Engineering, City University of Hong Kong, Tat Chee Avenue, Kowloon Tong, Hong Kong 999077, China; alvinlai@cityu.edu.hk; 3 Department of Aerospace Engineering, Khalifa University of Science and Technology, Abu Dhabi P.O. Box 127788, United Arab Emirates; kin.liao@ku.ac.ae; 4 Department of Physiology and Immunology, Khalifa University of Science and Technology, Abu Dhabi P.O. Box 127788, United Arab Emirates; peter.corridon@ku.ac.ae; 5 Healthcare Engineering Innovation Center, Khalifa University of Science and Technology, Abu Dhabi P.O. Box 127788, United Arab Emirates; 6 Center for Biotechnology, Khalifa University of Science and Technology, Abu Dhabi P.O. Box 127788, United Arab Emirates; 7 Department of Chemical and Biomolecular Engineering, University of Pennsylvania, Philadelphia, PA 19104, USA; graves@seas.upenn.edu; 8 Department of Biomedical Engineering, Khalifa University of Science and Technology, Abu Dhabi P.O. Box 127788, United Arab Emirates

**Keywords:** fluorescence recovery after photobleaching, biomolecules, polymers, transport, reaction, bio-interfaces, biophysical techniques

## Abstract

Among the new molecular tools available to scientists and engineers, some of the most useful include fluorescently tagged biomolecules. Tools, such as green fluorescence protein (GFP), have been applied to perform semi-quantitative studies on biological signal transduction and cellular structural dynamics involved in the physiology of healthy and disease states. Such studies focus on drug pharmacokinetics, receptor-mediated endocytosis, nuclear mechanobiology, viral infections, and cancer metastasis. In 1976, fluorescence recovery after photobleaching (FRAP), which involves the monitoring of fluorescence emission recovery within a photobleached spot, was developed. FRAP allowed investigators to probe two-dimensional (2D) diffusion of fluorescently-labelled biomolecules. Since then, FRAP has been refined through the advancements of optics, charged-coupled-device (CCD) cameras, confocal microscopes, and molecular probes. FRAP is now a highly quantitative tool used for transport and kinetic studies in the cytosol, organelles, and membrane of a cell. In this work, the authors intend to provide a review of recent advances in FRAP. The authors include epifluorescence spot FRAP, total internal reflection (TIR)/FRAP, and confocal microscope-based FRAP. The underlying mathematical models are also described. Finally, our understanding of coupled transport and kinetics as determined by FRAP will be discussed and the potential for future advances suggested.

## 1. Introduction

The rapid evolution of “omics” technology (genomics, proteomics, transcriptomics, and metabolomics) has led to knowledge about the identities and roles of molecules in cellular signalling pathways, tissue homeostasis, and organ functions [1]. The progress of omics-based experiments and advanced bioinformatic tools, artificial intelligence, and data science has led to the exponential increase in “Big Data” [2]. Such large and diverse sets of information create a bottleneck in correlating the conclusions drawn from large-scale data analytics with experimentally validated functional roles (e.g., binding kinetics, diffusion rates, etc.) of molecular targets in drug discovery [3].

In general, most cellular functions are executed by a series of highly synergistic signalling pathways involving molecular recognition, catalytic reaction, and phase partition of a vast number of biomolecules under molecular transport within specialized compartments, e.g., the nucleus [4]. In addition, the intricate interplay between transport processes and reaction kinetics plays a pivotal role in the embryogenesis, organ morphogenesis, tissue homeostasis, lymphatics, haemodynamics, paracellular permeability, tumour angiogenesis, and tumour metastasis [5]. As a result, a thorough understanding of key quantifiable physical parameters in biomolecular transport and kinetics of emerging molecular targets is critical to the translation of key research findings into new applications in drug discovery [6]. For instance, the design of molecular imaging probes, development of artificial organs, screening of structure-based inhibitors against inflammatory responses, overcoming the blood-tissue barrier in drug delivery, engineering tissue morphogenesis, and finding effective antiviral drugs could benefit from the research in cell and molecular biophysics [7]. Better understanding of basic physical and chemical rate processes will help determine the roles of key biomolecular targets in cell signalling pathways and physiological systems [8].

Even with the explosion of biological “Big Data”, there is a need for robust biophysical techniques to probe key targets at physiological length scales and in cellular microenvironments [9]. For many years, a critical area of biomedical engineering has been cancer detection, the effects of the tumour microenvironment, and how to target specific drugs [10]. For instance, the plasma pharmacokinetics and cellular pharmacodynamics of chemotherapeutic agents are shown to be directly affected by the interstitial transport [11,12,13,14], tumour angiogenesis [15], lymphatic clearance [16], and binding activity against cancer-specific markers [17,18,19]. In general, it has been known that significant resistance to mass transport and occurrence of non-specific binding encountered by therapeutic molecules causes indiscriminate drug distribution, poor penetration, inefficient cellular uptake, and limited success in cancer detection and treatment [20,21].

New microscopic methods have been developed to study biomolecule dynamics in complex media [22]. Since the 1980s, fluorescence relaxation has been used with the epifluorescence microscope to probe the diffusion of fluorescently labelled molecules, including dextran, bovine serum albumin, and various antibodies in both normal and cancerous tissues, extracellular matrices, and hydrogels [12,23,24,25,26,27,28]. One group developed an in vivo imaging tool known as the rabbit ear chamber, a transparent plastic device implanted on the ear of a male rabbit for direct and continuous observation of the live tissues [29]. Generally, the classical fluorescence relaxation technique is based on the measurement of spatio-temporal concentration profiles following a step change in concentration [30]. However, such a method often leads to errors in complex media, such as gel phase, due to limited spatial resolution and external perturbations during application of the step change [14,31,32]. Most importantly, the main drawback of the fluorescence relaxation technique or an improved approach, such as laser scanning confocal microscopy (LSCM), is the inability to distinguish unambiguously between convective, diffusive, and kinetic processes in complex media, such as tissues and biofilms [33,34,35].

In addition to simultaneous transport and reaction in complex media within physiological systems, other processes involving interfacial regimes in the cell organelles, drug delivery vesicles, biomaterials, adherent cells, RNA-protein complexes, and plasma membranes have been studied [36]. Biomolecular transport and kinetics at such biological and biomimetic interfaces are critical to receptor-mediated cell signalling, gene regulation, tumour cell metastasis, and applications in biotechnology, such as immobilized enzyme reactors, biofluidic assays, and biofilm eradication [37,38]. More specifically, biological signalling molecules, such as hormones, engage in specific binding with complementary targets at interfacial regions in plasma membranes, cytoskeletal networks, nuclear matrices, nuclear membranes, endocytic vesicles, ribosomes, etc. [39,40,41]. In biotechnology, biomolecules are similarly involved in adsorption, diffusion, and reaction at liquid-solid interfaces in biomaterials, separation media, MEMS devices, and biosensors, as well as on solid enzyme and affinity supports, etc. [42,43,44]. Frequently, such interfacial processes begin with physical adsorption of biomolecules from solution onto a solid phase, such as a polymeric membrane or porous material followed by surface diffusion and/or reaction, mimicking the biophysical events in cellular systems [45,46]. Since the 1980s, several biophysical techniques have been developed to probe the transport, adsorption, and binding kinetics of biomacromolecules at liquid-solid interfaces [47]. For example, ellipsometry and chiral sum frequency generation (SFG) spectroscopy have been applied to probe surface concentrations and interfacial conformations of adsorbed molecules, respectively [48,49].

Up until now, measuring the transport and reaction parameters in live cells, animal models, and biomimetic interfaces has remained challenging because most conventional techniques are invasive and thus are inapplicable to real-time measurement of key parameters, e.g., ligand binding to G-protein coupled receptors and solute transport in lymphatic nodes [50,51]. For instance, surface plasmon resonance (SPR), an established bioanalytical technique that is well known for probing the association/dissociation kinetics of biomolecules on their immobilized counterparts at interfaces in laminar flow, is only applicable to gold thin films coated with proteins or other biomolecules [52]. Indeed, advancements in drug target identification, intracellular signalling, cancer therapeutics, protein expression, drug delivery, and other research calls for new techniques that are applicable to a wide spectrum of physiochemical properties, such as hydrophobicity and size [53]. Such new methods will be needed, for example, in tumour microenvironments [54].

Among common biophysical techniques, FRAP has emerged as the most versatile and economical approach for studying coupled transport/reaction processes under various experimental configurations as shown in Table 1 (with the following abbreviations: * Signal to noise: S/N; Region of Interest: ROI. # Fluorescence Cross-Correlation Spectroscopy: FCCS). In comparison with FRAP, single particle tracking is not accurate for the measurement of fast-moving molecules due to the limitation of spatial-temporal resolution in the measured path of individual fluorescence particles [55]. Moreover, the application of fluorescence correlation spectroscopy is impaired by the lack of universal mathematical models for extracting key transport/kinetics parameters in more complex systems [56]. At the same time, two emerging biophysical techniques, including SPR sensor and stochastic optical reconstruction microscopy, impose higher instrumentation cost and provide less versatility to study diversified experimental systems compared to FRAP [52]. Interestingly, the recent advancement of fluorescence cross-correlation spectroscopy has enabled the quantitation of association between liposome and DNA, but the technique is inapplicable to probe immobilized molecules [57,58,59].

FRAP was first developed by Axelrod and Webb in 1976 and was based on the use of a single focused laser beam both for photobleaching and for monitoring fluorophore at high and low laser powers, respectively. Measuring the replenishment of intact fluorophore within the bleached spot leads to a value for the diffusion coefficient in solution (for example, a molecule tagged with rhodamine 6G) [71]. Since the 1980s, classical FRAP, as described previously, has been extensively exploited in fundamental research on membrane biophysics and physiological transport. This is referred to as epifluorescence spot FRAP hereafter [72,73,74]. During the 1990s, Jain et al. systemically explored the applications of epifluorescence spot FRAP to the combined diffusion and convection of biomacromolecules in normal and neoplastic tissues. Their method overcomes the drawbacks of conventional techniques, such as fluorescence relaxation [13,75]. Their group discovered that the diffusion coefficients obtained from epifluorescence spot FRAP is higher than that obtained by relaxation methods and is closer to that found in aqueous solution because FRAP measures diffusion primarily in the fluid phase of the interstitium [17,76]. Additionally, FRAP proved to be a promising technique for measuring diffusion and convective movements simultaneously in animal models in vivo, such as dorsal skinfold chambers [77]. Recently, the accuracy of FRAP measurement, which was validated in various physiological systems, has been further improved by detecting fluorescence with a charged coupled device (CCD) camera in conjunction with circular averaging of each image and spatial frequency analysis of the averaged radial data [78].

In the areas of biomaterials and biosensors, the challenge of characterizing biomolecules at solid-liquid interface has been overcome by the development of total internal reflection (TIR) fluorescence, which has emerged as an effective technique for detecting a variety of important parameters. These include the adsorption isotherm, interfacial structures, spatial distributions of molecules, and binding equilibria of molecules either weakly associated with or strongly bound to materials surfaces [79,80]. In TIR, a thin layer of surface-associated illumination (the evanescent wave) about 100 nm in thickness penetrates the liquid medium adjacent to the reflective surface [81,82]. Thus, the evanescent electromagnetic field excites fluorescent molecules immediately adjacent to the liquid-solid interface, with minimal interference from overlying molecules in solution [83]. When TIR produced by an Ar-ion laser source is coupled with FRAP, the integrated approach emerges as a very powerful technique for studying coupled diffusion and reaction kinetics [84]. In summary, a low intensity laser beam with an attenuated power of about 5–50 μw allows direct monitoring of the fluorescence intensity following bleaching by a laser beam with a power between 0.2 and 0.5 W [85]. As fluorescently tagged molecules diffuse back into the bleached region either from adjacent unbleached regions of the surface or from solution, their movement is detected. The resulting data is relevant to biomaterials research, bioseparations, biosensors, etc. Important parameters can be found by fitting the fluorescence recovery data with appropriate mathematical models [86].

More recently, LSCM (a confocal microscope equipped for laser scanning, Carl Zeiss, Jena, Germany), using a scanning laser beam both for illumination and photobleaching, has opened the possibility of performing FRAP measurements in most commercially available models of confocal microscopes, extending this valuable technique to more potential users [87]. One of the main advantages of the FRAP-based technique is that it provides a geometrically and temporally well-defined system. This makes it amenable to advanced mathematical analysis and numerical simulations to model tightly coupled transport/reaction processes [88]. For instance, a few groups have developed mathematical models for protein binding reactions between antibodies and tumour antigen-coated beads. The mathematical results agreed well with the experimental data [17,89]. In another case, the analytical solution of a diffusion-reaction model in the diffusion-limited regime fit the FRAP data very well for fluorescently labelled Concanavalin A in solution and Mannose immobilized on Sepharose beads [20].

Complex and tightly coupled transport-reaction processes are commonly found in cellular dynamics and interfacial biophysics, and these need to be unravelled. Despite the rapid development of FRAP techniques, the accurate decoupling of biophysical parameters within complex physiochemical environments remains challenging. With the superior performance of a highly sensitive and ultrafast CCD camera, Spatial Fourier Transform analysis has been successfully applied to correlate a time series of FRAP images with diffusive processes in a light scattering medium and with anomalous diffusion in a complex fluid [90,91]. Recent studies have shown that advanced mathematical modelling plus FRAP can decouple biomolecular transport and kinetics in highly intricate biochemical and physiological systems [92]. In this work, the authors provide a holistic review on instrumentation development, mathematical modelling, and recent applications of fluorescence recovery after photobleaching (FRAP). The advantages and disadvantages of FRAP, as well as advanced applications, will be explored.

## 2. FRAP Theory and Data Analysis

To date, FRAP measurement of biological membranes, immunological reactions, the interstitial space, cancerous tissue, bone matrix, interfacial enzymatic reactions, highly scattering media, polymer substrates, and crosslinked hydrogels, have been reported. FRAP experimentation must be accompanied by mathematical modelling to accurately reveal the key parameters involved in convective flow, bulk and surface diffusion, chemical and physical adsorption dynamics, as well as values for the partition coefficients. Thus, the advancement of FRAP hinges on the application of mathematical models and quantitative analysis to a temporal record of fluorescence intensity recovery after photobleaching, to determine key biophysical parameters [93]. FRAP measurements can be made in three configurations: epifluorescence, total internal reflection, and confocal modes [89,94].

### 2.1. Epifluorescence Spot Photobleaching

**Figure 1:**
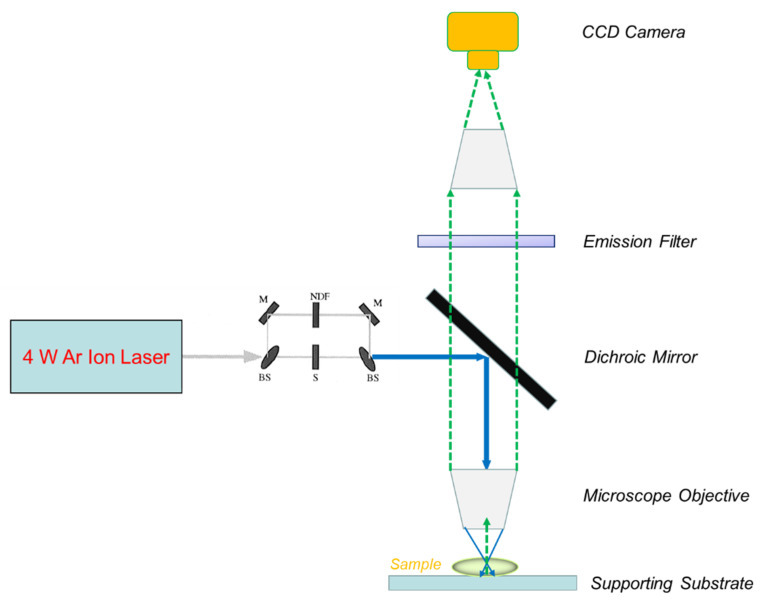
The general experimental setup of epifluorescence spot FRAP based on a high-power argon laser, upright microscope, and CCD camera. Beam Splitter: BS; Dichroic Mirror: DM; Mirror: M; Shutter: S; Neutral Density Filler: NDF.

Axelrod et al. pioneered the development of epifluorescence spot FRAP in 1976 by applying a 1 W Argon ion laser for both photobleaching and monitoring of rhodamine 6 G (a typical fluorescent tag) in water [71]. Figure 1 shows a modern setup of epifluorescence spot FRAP incorporating a powerful 4 W laser with a Gaussian intensity profile into a fluorescence microscope with a highly sensitive CCD camera for low light detection. Epifluorescence spot FRAP uses a beam-splitting module to divide the main laser beam into one high-power laser beam (controlled by a shutter) and an attenuated laser beam, both of which converge at the same spot on the sample under the microscope objective (Figure 1).

The three-dimensional (3D) geometry of the laser intensity profile used in FRAP directly dictates the concentration profiles of fluorescently labelled biomacromolecules (“fluorophore”) immediately after photobleaching and the recovery pattern of this profile at different time points after photobleaching as molecules diffuse back into the region [72]. The Gaussian intensity profile *I(r)* is described by a 3D radially symmetrical decay function as follows:

(1) $$I\left(r\right)=\left(\frac{2P_{0}}{\pi w^{2}}\right)e^{-\frac{2r^{2}}{w^{2}}}$$

where *r* is the radical distance from the centre of the Gaussian light profile, *w* is the half-width (the distance to achieve *e^−^*^2^ maximum laser intensity), and *P*_0_ is the total laser power. In a typical spot FRAP experiment, the intense laser beam with Gaussian intensity profile (from Equation (1)) at fixed wavelength (e.g., 488 nm) briefly illuminates the region of interests through the optical path to bleach the fluorophore, leading to the formation of an initial concentration profile of fluorophore (unbleached) immediately after photobleaching (at *t* = 0) as follows:

(2) $$C\left(r,0\right)=C_{0}e^{-\propto TI\left(r\right)}$$

where *C*_0_ is the uniform initial concentration of fluorophore before photobleaching, *T* is the duration of photobleaching carried by the high-powered laser beam, and −αI(r)  is the rate constant for the first order irreversible reaction involving the conversion of active fluorescent tag into its inactivated counterpart. The value of αTI(0)  is defined as the bleaching parameter *K* [82]. After the establishment of an initial concentration profile of unbleached fluorophore (Equation (2)), the concentration gradient at the boundary between the bleached region and the unbleached bulk phase will relax through lateral diffusion and bulk flow as summarized in the following equation:

(3) $$\frac{\partial C\left(r,t\right)}{\partial t}=D\nabla^{2}C\left(r,t\right)-V_{0}\left[\frac{\partial C\left(r,t\right)}{\partial x}\right]$$

where *D* is the diffusion coefficient, *V*_0_ is the uniform flow velocity in the x-direction, and the boundary condition is concentration outside the illumination region as shown by $C\left(\infty ,t\right)=C_{0}$.

**Figure 2:**
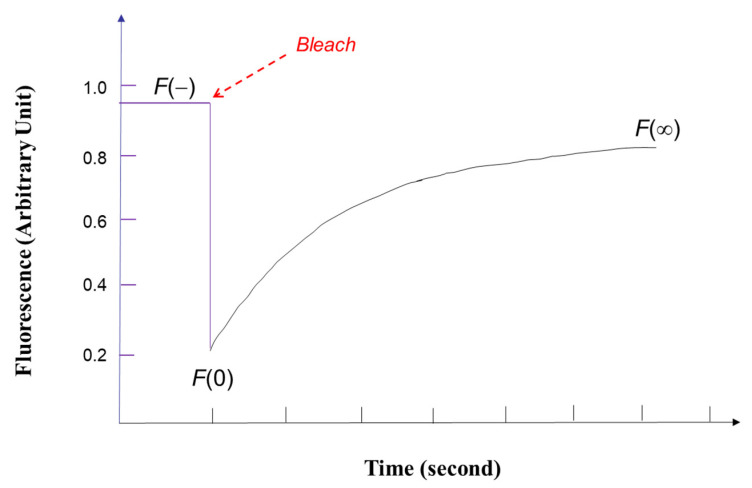
The fluorescence intensity measured by a CCD camera or PMT before and after photobleaching during FRAP experiments.

In spot FRAP, the main experimentally measured parameter is the integrated fluorescence intensity *F_k_*(*t*) collected from the region of sample illumination by a CCD camera or photomultiplier tube (PMT), which is typically plotted as a function of time (Figure 2) and can be conveniently described by the following equation:

(4) $$F_{K}\left(t\right)=\frac{q}{A}\int I\left(r\right)C_{K}\left(r,t\right)d^{2}$$

where *q* is the quantum efficiency of fluorescence excitation and emission, *A* is the attenuation factor of the total laser power for monitoring the fluorescent labelled biomolecules sample after photobleaching, *I(r)* is as given above, and *C_K_*(*r, t*) is the spatial-temporal concentration of fluorophores (directly dependent on *K*) driven by diffusion and bulk flow as governed by Equation (3). By applying the initial condition of unbleached fluorophores (Equation (2)) into Equation (4), the fluorescence intensity immediately after photobleaching with the Gaussian laser beam can be obtained as follows:

(5) $$F_{K}\left(0\right)=\left(\frac{qP_{0}C_{0}}{A}\right)K^{-1}\left(1-e^{-K}\right)$$

which is independent of the mechanism of molecular diffusion and bulk flow.

It can be further shown that the fluorescence intensity before bleaching *F*^0^ can be defined by $\frac{qP_{0}C_{0}}{A}$. Fourier transformation incorporating the given boundary conditions can be applied to solve Equation (3) [71]. Fourier transformation of *C_K_*(*r, t*) is then applied to Equation (4), leading to the solution for fluorescence intensity after bleaching $F_{K}\left(t\right).$ This function is governed only by diffusion (without bulk flow: *V*_0_ = 0) and is given by:

(6) $$F_{K}\left(t\right)=F^{0}vK^{-v}\int_{0}^{K}u^{v-1}e^{-u}du$$

where $u\equiv Ke^{-\frac{2r^{{\;}^{\prime }2}}{w^{2}}}\;$, *r′* is the Fourier transform space of *r*, $v\equiv {\left(1+\frac{2t}{\tau_{D}}\right)}^{-1}$, and $\tau_{D}\equiv \frac{w^{2}}{4D}$. The solution of Equation (6) for large values of *K* is approximated as:

(7) $$F_{K}\left(t\right)=F^{0}vK^{-v}\Gamma (v)$$

where *Γ*(*v*) is a gamma function. The series solution of Equation (7) for fitting FRAP recovery data under Brownian diffusion can be expanded as follows:

(8) $$F_{K}\left(t\right)=\frac{F_{0}+F_{\infty }\left(\frac{t}{\tau_{D}}\right)}{1+\frac{t}{\tau_{D}}}$$

where *F_0_* = $\;F_{K}\left(0\right)$ is the fluorescence intensity immediately after photobleaching, $F_{\infty }$ is the fluorescence intensity at infinite time after photobleaching, and a mobile fraction *R,* which describes the amount of exchange between bleached and unbleached fluorescent biomolecules (inside and outside the region of interest respectively), can be defined by $\frac{F_{\infty }-F_{0}}{F^{0}-F_{0}}$. *D* is determined by fitting the experimental FRAP data with Equation (8).

Although epifluorescence spot FRAP has emerged as a reliable technique for probing diffusion, the use of the spatially averaged fluorescence intensity $F_{K}\left(t\right)$ has been shown to be inaccurate for distinguishing between diffusion and convection unless the highly coupled transport process is convection dominated [13]. Measurement of the spatio-temporal concentration profile of unbleached fluorophores is necessary for simultaneously measuring the diffusion coefficient and flow velocity. Jain and co-workers have modified the first-generation instrumentation by replacing the attenuated laser beam and photomultiplier tube with a super-pressurized mercury lamp and silicon intensified target (SIT) camera, respectively, to probe the concentration profiles of unbleached fluorophores [34]. The mercury lamp provides uniform illumination rather than the laser’s gaussian illumination profile. With this new experimental setup, the fluorescence intensity at any location in the field of view of the SIT camera is proportional to the concentration of fluorophore, e.g., $F_{K}\left(t\right)=C\left(t\right)$. Based on the modified instrumentation, a theoretical model on the convection -diffusion process of unbleached fluorophores from the region outside the Gaussian bleached spot (generated from the Gaussian beam of the high-power laser) into the bleached region can be described by the following equation [33]:

(9) $$\frac{\partial C}{\partial t}=D\frac{\partial^{2}C}{\partial x^{2}}+D\frac{\partial^{2}C}{\partial y^{2}}-v_{x}\frac{\partial C}{\partial x}-v_{y}\frac{\partial C}{\partial y}$$

where *C* is the fluorophore concentration at any position (*x*, *y*) in the field of view of the epifluorescence microscope; *D* is the lateral diffusion coefficient of fluorophore; $v_{x}$ and $v_{y}$ are the *x* and *y* components of uniform 1-D convective velocity, respectively, while *v* is under the following boundary conditions:

(10) $$C\to C_{\infty }\;as\;x\to \infty \;;\;y\to \infty$$

(11) $$\frac{\partial C}{\partial x}=0\;at\;x=x_{0}\;;\;\frac{\partial C}{\partial y}=0\;at\;y=y_{0}\;$$

where $C_{\infty }$ is the concentration of the fluorophore far away from the bleached spot, and $\left(x_{0,\;}\;y_{0}\right)$ is the centre of Gaussian bleached spot.

By applying a Fourier Transform, Equation (9), under the given boundary conditions, was successfully solved as a function of time with the use of the Fourier Convolution Theorem as follows [33]:

(12) $$C\left(x,y,t\right)=C_{o}\left(t\right)+\left(C_{\infty }-C_{o}\left(t\right)\right)\left[1-e^{-\frac{2\left[{\left(x-x_{0}\left(t\right)\right)}^{2}+{\left(y-y_{0}\left(t\right)\right)}^{2}\right]}{R^{2}\left(t\right)}}\right]$$

where the spatio-temporal parameters of the convective-diffusion process be further defined as follows:

(13) $$C_{o}\left(t\right)=C_{o}\left(0\right)+\left(C_{\infty }-C_{o}\left(0\right)\right)\left(\frac{8Dt}{R^{2}\left(t\right)}\right)$$

(14) $$R^{2}\left(t\right)=R^{2}\left(0\right)+8Dt$$

(15) $$x_{0}\left(t\right)=x_{0}\left(0\right)+v_{x}t$$

(16) $$y_{0}\left(t\right)=y_{0}\left(0\right)+v_{y}t$$

where $C_{o}\left(0\right)\;$ is the initial concentration of the fluorophore at the Gaussian bleach centre, and *R*(*t*) is the size of the Gaussian bleach spot at any time *t* after photobleaching. As the Gaussian laser beam creates a photobleached spot that has a Gaussian profile, the initial condition of the concentration of fluorophores can be written as:

(17) $$C\left(x,y,0\right)=C_{o}\left(0\right)+\left(C_{\infty }-C_{o}\left(0\right)\right)\left[1-e^{-\frac{2\left[{\left(x-x_{0}\left(0\right)\right)}^{2}+{\left(y-y_{0}\left(0\right)\right)}^{2}\right]}{R^{2}\left(0\right)}}\right]$$

where *R*(0) is the initial radius of the photobleached spot (40 μm), and $C_{o}\left(0\right)$ is obtained by calculating the initial concentration (*t* = 0) at any given distance from the centre of the bleached spot. By fitting fluorescence intensity (identical to concentration in this experimental setup) as a function of distance from the photobleached centre at various time points from Equation (12) after photobleaching using a modified Newton’s non-linear parameter estimation method, the four unknowns *x*_0_, *y*_0_, *C*_0_, and *R*_0_ at each time point can be obtained [13]. A non-linear regression of *C*_0_ versus time yields the value of the diffusion coefficient (*D*) from Equation (13). The two components of the convective velocity can be determined by fitting *x*_0_ and *y*_0_ against time with Equation (15) and Equation (16) by linear regression analysis. The above analysis has been applied to convection and diffusion analysis in relatively thin tissue samples (thickness < 40 μm) without chemical reaction.

The two mathematical models of epifluorescence spot FRAP were successfully applied for the analysis of convective-diffusive processes, assuming that molecular binding or reaction was absent. To probe coupled transport–reaction processes, a more sophisticated mathematical model was developed by Kaufman and Jain for modelling the coupled mass transport and binding kinetics between molecules, such as antibodies (fully mobile in the liquid phase in the absence of binding) and tumour antigens (immobilized on 1.6 μm Sepharose beads) in bulk solution, as shown by the following reaction:

(18) $$C_{1i}+Ag↔C_{2i}$$

The coupled diffusion and reaction can be described by the following mathematical equation, assuming that there is a uniform distribution of binding sites on a bead, that the bleached molecules are still biologically active, there is no convection, and that the bound complex is immobile:

(19) $$\frac{\partial C_{1i}}{\partial t}=D\nabla^{2}C_{1i}-k_{1}C_{1i}Ag+k_{-1}C_{2i}$$

(20) $$\frac{\partial C_{2i}}{\partial t}=k_{1}C_{1i}Ag-k_{-1}C_{2i}$$

where *C*_1*i*_ is the concentration of mobile biomacromolecule (and the subscript *i* is b for bleached or a for unbleached), *C*_2*i*_ is the concentration of immobile biomacromolecule, *Ag* is the concentration of vacant binding sites, and *Ag*_0_ is the total concentration of binding sites (*Ag* + *C*_2*i*_). Most importantly, the same coupled diffusive-reactive process modelled by Equations (19) and (20) is applicable to both unbleached and bleached fluorescently tagged molecules. As the system has a cylindrical geometry and is only dependent on the radial direction (*r*), the differential equation of molecular diffusion in a cylindrical geometry can be applied as follows:

(21) $$\nabla^{2}C_{1i}=\frac{1}{r}\frac{\partial }{\partial r}\left(r\frac{\partial C_{1i}}{\partial r}\right)$$

By using a super-pressurized mercury lamp that provides uniform illumination across the field of view, the detected fluorescence intensity is directly correlated with the concentration of unbleached fluorescently labelled species [17]. The initial concentration profile of the sum of bleached mobile and immobile fluorescently tagged biomacromolecules (which are assumed to be at chemical equilibrium with each other) immediately after photobleaching (at *t* = 0) is directly related to the Gaussian intensity profile of the laser beam as follows:

(22) $$C_{1a}+C_{2a}=C_{Ta}=C_{TB}+\left(C_{TU}-C_{TB}\right)\left[1-exp\left(\frac{-2r^{2}}{R_{0}^{2}}\right)\right]$$

where $C_{Ta}$ is the total concentration of fluorescently active molecules (mobile and immobile) at a given location in the bleached profile, $C_{TU}$ is the total concentration of fluorescent species (mobile and immobile) in the region far away from the bleached spot (r→∞), $C_{TB}$ is the initial concentration of these species at the centre of the bleached spot (*r* = 0 and *t* = 0), and *R*_0_ is the initial Gaussian radius of the bleached spot (*t* = 0). By assuming that a chemical equilibrium is established between immobile and mobile species, the following boundary conditions can be written:

(23) $$\frac{\partial C_{1i}}{\partial r}=\frac{\partial C_{2i}}{\partial r}=0\;at\;r=0\;and\;at\;r\to \infty \;$$

(24) $$\frac{k_{1}}{k_{-1}}=\frac{C_{2a}}{C_{1a}\left(Ag_{0}-C_{2a}-C_{2b}\right)}$$

(25) $$\frac{k_{1}}{k_{-1}}=\frac{C_{2b}}{C_{1b}\left(Ag_{0}-C_{2a}-C_{2b}\right)}$$

To establish the correlation between fluorescence recovery and physical parameters, several dimensionless terms are defined as follows:

(26) $$\theta_{1a}=\frac{C_{1a}}{C_{TU}-C_{TB}}\;;\theta_{2a}=\frac{C_{2a}-C_{TB}}{C_{TU}-C_{TB}}\;;\;\theta_{1b}=\frac{C_{1b}}{C_{TU}-C_{TB}}\;;\;\theta_{2a}=\frac{C_{2b}-C_{TB}}{C_{TU}-C_{TB}}\;$$

(27) $$z=\frac{r}{R_{0}}\;;\;\tau =\frac{Dt}{R_{0}^{2}}\;;Da=\frac{R_{0}^{2}k_{1}Ag_{0}}{D}\;;\;\alpha =\frac{C_{TB}}{Ag_{0}}\;;\;\beta =\frac{k_{1}Ag_{0}}{k_{-1}}\;;\;\gamma =\frac{C_{TB}}{C_{TU}-C_{TB}}\;$$

**Figure 3:**
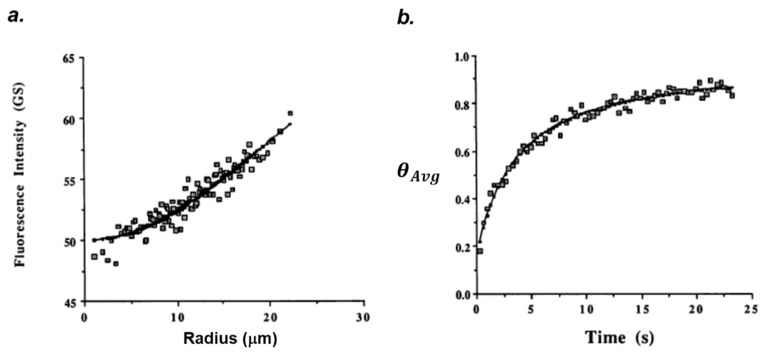
Photobleaching recovery for (**a**) fluorescence intensity ($I_{TA}\propto C_{TA}$) against *r* plotted from *t* = 0, immediately after photobleaching is fitted with Equation (22) for determining the value of *R*_0_ and $I_{TB}$. (**b**) *D_eff_* is determined by fitting the dimensionless average fluorescence intensity data $\theta_{Avg}$ against time by fitting to Equation (31) and β is determined by measuring *D* in nonbinding system. Reprinted with permission from [95]. Copyright 1991, Elsevier.

By rearranging the fluorescence recovery equations with dimensionless average fluorescence intensity, this mathematical model of antibody-antigen binding results in four nonlinear, coupled partial differential equations (PDEs) describing each of the four types of molecules, including active fluorophore tagged antibody (mobile), bleached fluorophore tagged antibody (mobile), active fluorophore tagged antibody (immobilized), and bleached fluorophore tagged antibody (immobilized). The two equations for active fluorophore tagged antibody (mobile) and active fluorophore tagged antibody (immobilized) are:

(28) $$\frac{\partial \theta_{1a}}{\partial \tau }=\frac{1}{z}\frac{\partial }{\partial z}\left(z\frac{\theta_{1a}}{\partial z}\right)-Da\left\{\theta_{1a}\left[1-\alpha \left(\frac{\theta_{2a}+\theta_{2b}}{\gamma }+1\right)\right]-\frac{\theta_{2a}+\gamma }{\beta }\right\}$$

(29) $$\frac{\partial \theta_{2a}}{\partial \tau }=Da\left\{\theta_{1a}\left[1-\alpha \left(\frac{\theta_{2a}+\theta_{2b}}{\gamma }+1\right)\right]-\frac{\theta_{2a}+\gamma }{\beta }\right\}$$

where $\theta_{1i}$ and $\theta_{2i}$ are the dimensionless concentration of fluorescently active mobile and immobilized antibody, respectively. *Da* and τ  represent the Damkohler number and dimensionless time, respectively. The equations for the two additional species are in similar forms, such as Equations (28) and (29), and are not shown. The analytical solution of the four coupled differential equations can be obtained for two common types of antibody-antigen recognition, including diffusion-limited binding and reaction-limited binding [95]. Under the regime of diffusion-limited reaction (when Da≫1), the four coupled PDEs can be solved by Fourier Transformation followed by spatial integration to yield the dimensionless average fluorescence intensity $\left(\theta_{Avg}\right)\;$ as a function of time over a square region with a side *ξ* (cantered at the middle of beached spot) as follows:

(30) $$\theta_{Avg}=\frac{I_{Avg-}I_{TB}}{I_{TU-}I_{TB}}=1-\frac{\pi }{4u^{2}}erf^{2}\left(\frac{u}{\sqrt{1+8\tau }}\right)$$

where $u=\frac{\sqrt{2}}{R_{0}}\;;\;\tau =\frac{D_{eff}t}{R_{0}^{2}}\;;D_{eff}=\frac{D}{1+\beta }\;;\;\beta =\frac{k_{1}Ag_{0}}{k_{-1}}\;$. *I_Avg_* is the integrated average fluorescence at time t measured by a PMT or CCD camera from the bleached region during FRAP experiments. The fluorescent intensity ($I_{TA}\propto C_{TA}$) as a function of *r* plotted at *t* = 0, immediately after photobleaching, is used to fit Equation (22) to determine the values of *R*_0_ and $I_{TB}\;$ (Figure 3a). Second, *D_eff_* is determined by fitting $\theta_{Avg}\;$ against time and β is determined by measuring *D* in a nonbinding system (Figure 3b).

Under conditions of reaction limited binding (when Da≪1), any biologically active antibody (unbleached or bleached) exists in either the bound state (immobilized on antigen coated bead) or the unbound state (fully diffusive in the liquid phase) during the time course of FRAP measurements [13]. This assumption is valid when the rate of association or dissociation of the antibody/antigen complex is significantly lower than the rate of diffusion of antibody to the antigen-coated bead surface. The analytical solution of the reaction-limited binding regime results in the dimensionless average fluorescence intensity at the centre of the Gaussian photobleached spot as follows:

(31) $$\theta_{Avg}=\frac{I_{Avg-}I_{TB}}{I_{TU-}I_{TB}}=1-\frac{\pi }{4u^{2}}\left[\left(1-\varphi_{0}\right)erf^{2}\left(\frac{u}{\sqrt{1+8\tau }}\right)+\varphi_{0}erf^{2}u\right]$$

By fitting the experimentally measured the data of average fluorescence intensity in the photobleached spot against time, the values of the uncorrected immobile fraction (*ϕ_0_*) and diffusion coefficient (*D*) of antibody can be determined (as in Figure 3b).

It is well known that some of the labelled antibodies will become biologically inactive during interaction with the bead and fail to bind to the antigenic site. As a result, it is necessary to convert *ϕ*_0_ to represent only the fraction of biologically active antibodies that are immobile (*ϕ_c_*) following antibody–antigen binding on the bead as follows.

(32) $$\varphi_{c}=\frac{\varphi_{0}}{1-nr}=\frac{C_{2a}+C_{2b}}{Ab_{inc}}$$

where *nr* is fraction of total antibodies (both mobile and immobile) rendered biologically inactive, and *Ab_inc_* is the incubating concentration of biologically active antibody. Based on the definition of the equilibrium constant for antibody binding to the antigen-coated bead (*K_eq_*), *ϕ_c_* can be expressed as a function of *Ab_inc_*:

(33) $$\varphi_{c}=\frac{1}{2}\left\{\frac{1+K_{eq}\left(Ag_{0}+Ab_{inc}\right)}{K_{eq}Ab_{inc}}-{\left[{\left(\frac{1+K_{eq}\left(Ag_{0}+Ab_{inc}\right)}{K_{eq}Ab_{inc}}\right)}^{2}-\frac{4Ag_{0}}{Ab_{inc}}\right]}^{0.5}\right\}$$

and

(34) $$K_{eq}=\frac{C_{2a}+C_{2b}}{\left(Ab_{inc}-C_{2a}-C_{2b}\right)\left(Ag_{0}-C_{2a}-C_{2b}\right)}$$

where *Ag*_0_ is the total concentration of the binding sites (antigen). When *ϕ_c_* is measured as a function of biologically active antibody concentration (*Ab_inc_*), *K_eq_* and the concentration of the binding sites (*Ag*_0_) can be obtained by fitting the experimental data with Equation (33) through the application of a modified Newton’s nonlinear parameter estimation method [20].

### 2.2. Total Internal Reflection/Fluorescence Recovery after Photobleaching

**Figure 4:**
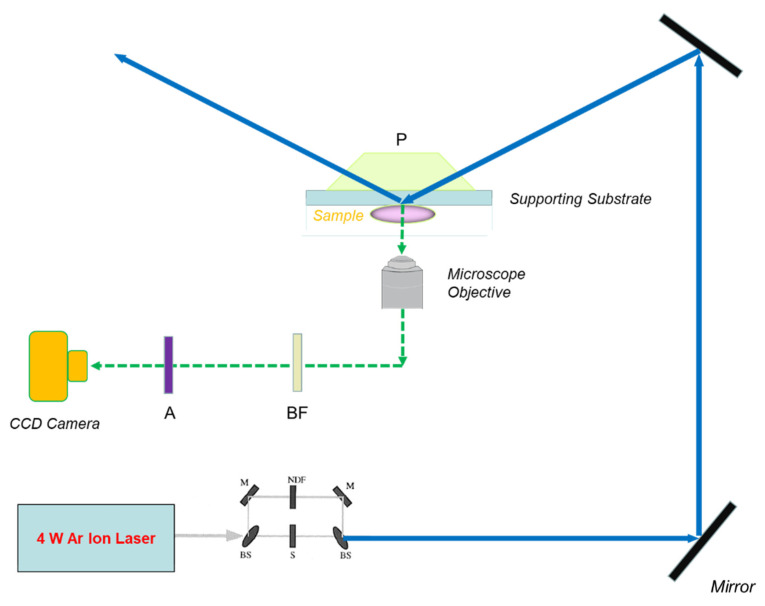
The overall optical train for the TIR/FRAP setup attached to an inverted microscope. The basic equipment is a 4 W argon ion laser, a low-light level-cooled CCD camera, an inverted microscope, and a computer for image capture and analysis. Beam Splitter: BS; Mirror: M; Dichroic Mirror: DM; Aperture: A; Prism: P; Shutter: S; Band Pass Filter: BF; Neutral Density Filler: NDF.

TIR occurs when a light beam travels through a transparent medium of a high index of refraction (e.g., a glass surface) to an interface of medium with lower index of refraction, such as an aqueous solution, and the incidence angle of the beam is larger than the critical angle *θ_C_*, which is dependent on the properties of the two transparent media forming the reflective interface:

(35) $$\theta_{c}=\sin^{-1}\left(\frac{n_{2}}{n_{1}}\right)$$

where *n*_1_ and *n*_2_ are the refractive indices of the glass and liquid, respectively. The totally internally reflected incident light creates an evanescent wave to excite fluorescently tagged molecules close to glass substrate [18,19]. The fluorescence signal is eventually collected by a microscope objective in a typical optical train of TIR/FRAP instrumentation, as shown in Figure 4 [36,37].

**Figure 5:**
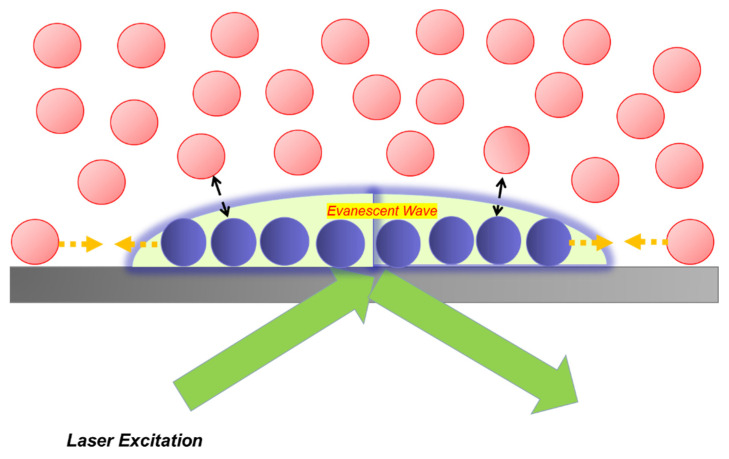
Total internal reflection (TIR) microscopy taps on the production of an evanescent wave, which specifically illuminates biomacromolecules (circles) close to the solid substrate (e.g., fused silica). Immediately after photobleaching, the fluorescent tag on the biomacromolecules were deactivated by the intense laser beam (purple circles) and lost the fluorescence signal to be detected by CCD camera or photomultiplier tube. Afterwards, the fluorescence signal recovers by the exchange of the bleached molecules inside the region of interest with those unbleached biomacromolecules (red circles) through adsorption/desorption kinetics and surface diffusion.

The evanescent wave is an electromagnetic field, as shown on Figure 5, which propagates parallel to the liquid/solid interface and penetrates a very small distance into the liquid medium. The intensity *I* of the evanescent wave decreases exponentially with the perpendicular distance *z*:

(36) $$I\left(z\right)=I_{0}e^{-\frac{z}{d}}$$

where *I*_0_ is the intensity at *z* = 0 and penetration depth *d* is a decreasing function of the angle of incidence *θ* and the ratio of refractive indices in the two media (*n*_1_/*n*_2_) at the interface (Figure 5):

(37) $$d=\frac{\lambda_{0}}{4\pi }{\left(n_{1}^{2}\sin^{2}\theta -n_{2}^{2}\right)}^{\frac{-1}{2}}$$

*d* must be smaller than the wavelength of light *λ*_0_, enabling the selective illumination of surface-bound or associated molecules on glass surface. Moreover, *d* is also independent of the polarization of the incident light. By focusing a coherent laser beam with the Gaussian profile in TIR mode at the reflective interface as mentioned above, an intensity profile with an elliptical Gaussian profile is generated as follows:

(38) $$I\left(r\right)=e^{-\frac{2x^{2}}{s^{2}}}e^{-\frac{2\gamma y^{2}}{s^{2}}}$$

where *x* and *y* are the components of a spatial coordinate *r*; γ is the size ratio (*y*:*x*) of the elliptical Gaussian intensity profile, and *s* is a characteristic dimension of the focused laser spot.

The basic theory of TIR spot/pattern photobleaching was developed by Axelrod and Thompson during the early 1980s [6,8,41,42]. Only the mathematical model for analysing the simplest type of TIR/FRAP measurement involving one type of fluorescently labelled molecules, which is in equilibrium with those specifically reacted, bound, or physically adsorbed on the surface sites, is illustrated herein.

(39) A(r,z,t)+S(r,t)↔C(r,t)

where *A* is the free diffusing molecules in solution (in 3D), which is tagged with fluorescence reagent; *S* is the adsorption or reactive (e.g., antigen) sites on the planar surface; *C* is the physically adsorbed or biochemically reacted biomacromolecules from the solution and can be mobile on the surface; *r* is the spatial coordinate on the surface and is measured from the centre of the spot; *z* is the perpendicular distance measured from the liquid/solid interface; *t* is a temporal parameter; and *k_+_*_1_ and *k_−_*_1_ are the adsorption (or forward reaction or binding) and desorption (backward reaction or dissociation) rate constants of the molecules, respectively. The equilibrium constant of this adsorption process is:

(40) $$K_{eq}=\frac{\bar{C}}{\left(\bar{A}\right)\left(\bar{S}\right)}=\frac{k_{+1}}{k_{-1}}$$

where $\bar{C}$, $\bar{A},\;$ and $\bar{S}$ are the equilibrium concentrations of the surface-bound biomacromolecules (through binding or adsorption), the free diffusing biomacromolecules in solution, and the surface-active sites, respectively.

The measurable experimental parameter of TIR/FRAP is the light signal generated from fluorophore excitations (F(t)) at the liquid/solid interface, which can be described by Equation (4) and experimentally detected by PMT or CCD camera [41,42]. In order to correlate the experimental fluorescence intensity with meaningful physical parameters, the concentration profiles of the adsorbed or surface-bound biomacromolecules *C*(*r*, *t*) and free diffusing molecules in solution *A*(*r*, *z*, *t*) must be expressed mathematically [41,42,61] as follows:

(41) $$\frac{\partial A}{\partial t}=D_{A}\nabla_{r,z}^{2}A$$

(42) $$\frac{\partial C}{\partial t}=D_{C}\nabla_{r}^{2}A+k_{+1}A_{Z\to 0}S-k_{-1}C$$

where *D_A_* and *D_C_* are the solution and surface diffusion coefficient, respectively. $\nabla_{r,z}^{2}$ and $\nabla_{r}^{2}\;$ represent the three- and two-dimensional Laplacians, respectively. $A_{Z\to 0}$ is the local bulk concentration of the free diffusing molecule in solution at the surface (*z* = 0). Equation (41) represents the diffusion of biomacromolecules from the solution to the liquid/solid interface. Equation (42) represents the surface diffusion of surface-bound molecules on the surface, and adsorption (or binding) to and desorption (dissociation) from the surface. An additional correlation is obtained by using Fick’s law in which the net diffusive flux to the reflective surface (glass) is equal to the difference between the number of molecules adsorbed (or forward binding) to that desorbed (or backward dissociation) per unit area per unit time as listed below:

(43) $$D_{A}{\left(\frac{\partial A}{\partial z}\right)}_{z\to 0}=k_{+1}A_{Z\to 0}S-k_{-1}C$$

In other words, a material balance on the adsorbing molecule. In order to solve for *F*(*t*) in TIR spot FRAP, a monotonically decreasing function is first defined as follows:

(44) $$G_{s}\left(t\right)=\bar{F}-F\left(t\right)$$

where the subscript *s* means spot FRAP. $\bar{F}$ is the equilibrium fluorescence intensity before photobleaching; *F*(*t*) is the fluorescence intensity at *t* > 0 after the application of a photobleaching pulse. The solution and surface concentrations are also normalized:

(45) $$A_{s}\left(r,z,t\right)=\bar{A}-A\left(r,z,t\right)$$

(46) $$C_{s}\left(r,t\right)=\bar{C}-C\left(r,t\right)$$

where *s* denotes TIR spot photobleaching and $\bar{A}$ and $\bar{C}$ are the equilibrium bulk and surface concentration of fluorescently tagged molecules (including both bleached and unbleached fluorophores), respectively, during the entire course of FRAP measurement. A(r,z,t) and C(r,t) are the equilibrium bulk and surface concentration of unbleached molecules, respectively, after application of the photobleaching pulse (starting from *t* = 0).

During the TIR/FRAP experiment, only the fluorescein-labelled biomolecules that are closed to the solid/liquid interface (z→0) and inside the focused laser spot are photobleached. The concentrations of unbleached molecules in the region far away from the bleached spot are equal to the equilibrium values ($\bar{A}$ and $\bar{C}$). As a result, the normalized concentration of bulk and surface bound concentration of biomacromolecules in regions far away from the bleached spot is equal to zero. By assuming the strong laser beam under TIR mode just bleaches the fluorophores that are close to the liquid/solid interface, the concentration of the unbleached molecules in the bulk solution is equal to the equilibrium bulk concentration $\bar{A}$ (at z→∞). Altogether, the boundary and initial conditions for the TIR/FRAP experiment are listed as follows:

(47) $${\left[C_{s}\left(r,t\right)\right]}_{\left|r\right|\to \infty }={\left[A_{s}\left(r,z,t\right)\right]}_{\left|r\right|\to \infty }={\left[A_{s}\left(r,z,t\right)\right]}_{z\to \infty }={\left[A_{s}\left(r,z,t\right)\right]}_{t\to 0}=0$$

Immediately after photobleaching at *t* = 0, the local concentration of the unbleached fluorescein tagged biomacromolecules adsorbed on the surface (*C*) is governed by the laser intensity profile (described by Equation (38)) during the bleaching reaction as follows [42,61]:

(48) $${\left[C_{s}\left(r,t\right)\right]}_{t\to 0}=\bar{C}\left(1-e^{-KI\left(r\right)}\right)$$

where *K* is the bleaching efficiency that depends on the bleaching power and duration as defined earlier for epifluorescence spot photobleaching. In contrast to the nonselective measurement of both the freely diffusive and surface-bound fluorophores in a transport-reaction system with epifluorescence spot FRAP, the normalized fluorescence signal (at *t* after bleaching) from TIR spot photobleaching is only dependent on the concentration change of surface-bound fluorophore as follows:

(49) $$G_{s}\left(t\right)=QI_{0}\int I\left(r\right)C_{s}\left(r,t\right)d^{2}r$$

and the initial condition is:

(50) $$G_{s}\left(0\right)=\bar{C}QI_{0}\int I\left(r\right)\left(1-e^{-KI\left(r\right)}\right)d^{2}r$$

In order to solve for Equation (49), Equations (41)–(43) were normalized in terms of *C_s_* and *A_s_*. Afterwards, those equations are Laplace transformed with respect to the normal to surface (*z* → *p*) and time (*t* → *ω*) and Fourier transformed with respect to the surface position vector (*r* → *q*). By expressing $A_{s}\left(q,p,\omega \right)$ in terms of other variables from the three normalized differential equations and inverse Laplace transforming from *p*-space back to *z* space, applying the boundary conditions (Equation (47)), and substituting ${\left[A_{s}\left(q,p,\omega \right)\right]}_{z\to 0}$ in terms of $C_{s}\left(q,\omega \right)$, the solution of the normalized form of Equation (43) yields the following expression of $C_{s}\left(q,\omega \right)$:

(51) $$C_{s}\left(q,\omega \right)=N\left(q,\omega \right){\left[C_{s}\left(q,t\right)\right]}_{t\to 0}$$

(52) $$N\left(q,\omega \right)=\frac{\sqrt{q^{2}D_{A}+\omega }+\frac{k_{+1}\bar{S}}{\sqrt{D_{A}}}}{\left(\;\omega +k_{-1}+q^{2}D_{A}\right)\sqrt{q^{2}D_{A}+\omega }+\left(\omega +q^{2}D_{C}\right)\frac{k_{+1}\bar{S}}{\sqrt{D_{A}}}}$$

To solve for $G_{s}\left(t\right)$, Equation (51) is substituted into Equation (49) with the initial condition Equation (48) using an inverse Lapse transform and an inverse Fourier transform as follows:

(53) $$G_{s}\left(t\right)=QI_{0}\int I\left(r\right)L_{\omega \to t}^{-1}F_{q\to r}^{-1}\left\{N\left(q,\omega \right)F_{i\to q}\left[\bar{C}\left(1-e^{-KI\left(r^{\prime }\right)}\right)\right]\right\}d^{2}r$$

By applying Parseval’s theorem, G(*t*) is rewritten as:

(54) $$G_{s}\left(t\right)=G\left(0\right)L_{\omega \to t}^{-1}\frac{\int {\left|I\left(q\right)\right|}^{2}N\left(q,\omega \right)d^{2}q}{\int {\left|I\left(q\right)\right|}^{2}d^{2}q}$$

where *I*(*q*) is the Fourier transform of *I*(*r*). The illumination area of a slightly focused Gaussian laser beam is usually large (with dimensions of 500 × 100 μm) and results in large characteristic distance *s*. By solving Equation (54) under the assumption that the bulk normal diffusion rate is significantly larger than the reaction rate or the desorption rate constant of biomacromolecules from the solid substrate, the reaction limited solution is:

(55) $$G_{s}\left(t\right)=G\left(0\right)e^{-k_{-1}t}$$

**Figure 6:**
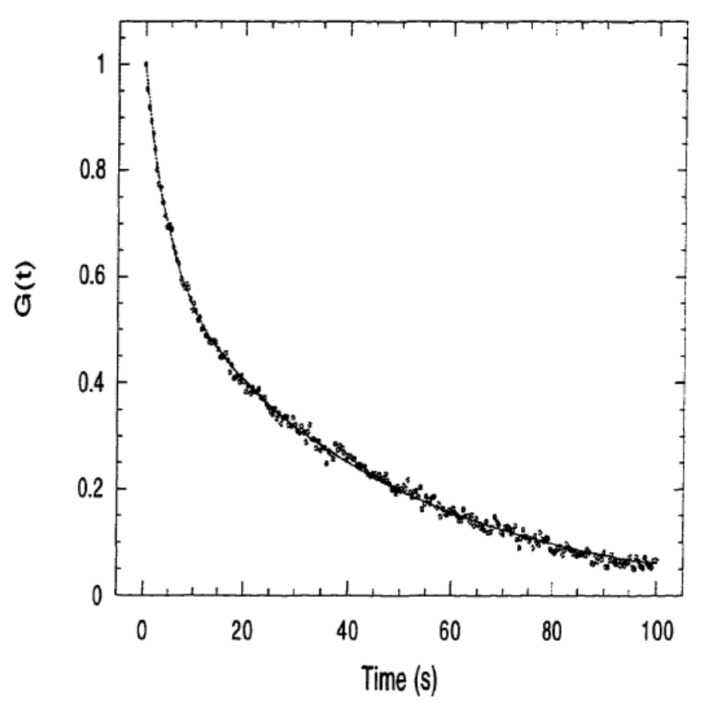
A conventional TIR/FRAP recovery curve (*F*(*t*) vs. time) of fluorescently tagged DNA in PBS on amino-coated glass which was fitted with two adsorption state model (extended version of Equation (49)). Reprinted/adapted with permission from [96]. Copyright 1997, Vincent Chan.

In this situation, the experimental recovery curve *G(t)* can be used to determine the kinetics of biomolecular binding or physical adsorption at the solid/liquid interface (Figure 6) [96]. An interesting situation is a reaction limited recovery of *G(t)* in the presence of two-dimensional diffusion on solid surface when a narrow beam of laser light is applied. *G(t)* can be expressed in terms of real space variables by applying Parseval’s theorem and the convolution theorem to yield the following solution:

(56) $$G_{s}\left(t\right)=G\left(0\right)\frac{e^{-k_{-1}t}}{\sqrt{\left(1+\frac{4D_{C}t}{s^{2}}\right)\left(1+\frac{4\gamma D_{C}t}{s^{2}}\right)}}$$

*G(t)* is the product of a simple exponential function that describes adsorption/desorption kinetics or forward/backward reaction kinetics and a factor that characterizes the surface diffusion rate.

### 2.3. Confocal FRAP

**Figure 7:**
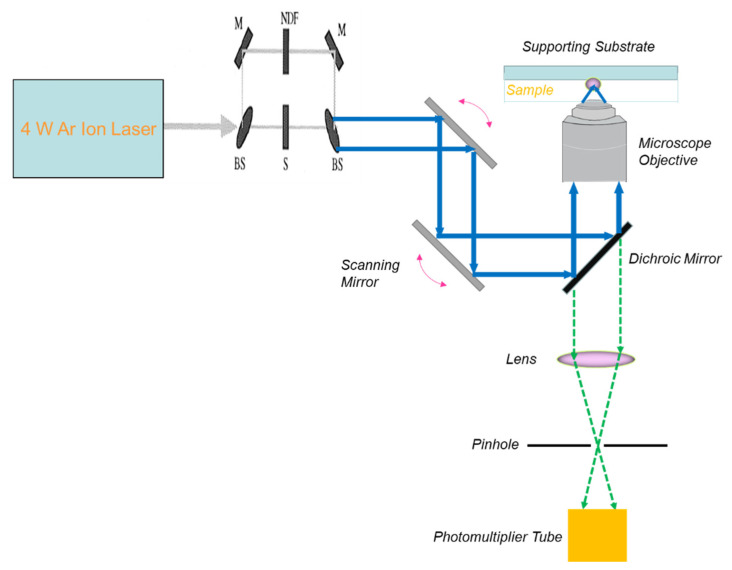
The overall optical train for the Confocal FRAP setup. Beam Splitter: BS; Aperture: A; Prism: P; Shutter: S; Neutral Density Filler: NDF.

More recently, LSCM, which provides an intense illumination for executing bleaching reaction of fluorophore, has emerged as an attractive platform for the incorporation of FRAP measurement of molecular diffusion of biomacromolecules in cells’ cytoplasm or organelles [92]. A typical setup of confocal FRAP, which is built on the experimental platform of LSCM, is shown in Figure 7.

By assuming that the size of the bleach spot is small compared to the cell dimension, the laser intensity profile for the confocal photobleaching is a Gaussian function under the condition of biomacromolecule diffusion in an infinite plane as defined in the following equation [56]:

(57) $$I\left(r\right)=\left(\frac{2I_{0}}{\pi r_{n}^{2}}\right)e^{\frac{-2\left(x^{2}+y^{2}\right)}{r_{n}^{2}}}$$

where *r_n_* is the nominal radius of the bleached spot, (*x*, *y*) is the spatial coordinate of the field of view, and *I*_0_ is the total laser power. For a pure diffusive process in solution phase, the spatio-temporal concentration of unbleached fluorescently tagged molecules (fluorophore) immediately after photobleaching (C(x,y,t)) is governed by Fick’s second law as follows:

(58) $$\frac{\partial C}{\partial t}=D\left(\frac{\partial^{2}C}{\partial x^{2}}+\frac{\partial^{2}C}{\partial y^{2}}\right)$$

By solving Equation (58), the fundamental solution of 2D-free diffusion of biomacromolecules from an infinite plane is [49]:

(59) $$\phi_{Dt}\left(x,y\right)=\frac{1}{4\pi Dt}e^{\frac{-\left(x^{2}+y^{2}\right)}{4Dt}}$$

At the same time, the initial concentration profile of bleached molecules *C*(*x*, *y*, 0) immediately following photobleaching (*t* = 0) by the Gaussian laser beam of LSCM is:

(60) $$C\left(x,\;y,\;0\right)=C_{i}\left(1-Ke^{\frac{-2\left(x^{2}+y^{2}\right)}{r_{e}^{2}}}\right)$$

where *C_i_* is the concentration of unbleached fluorophores before photobleaching, *K* is a bleaching-depth parameter, and *r_e_* is the effective radius of photobleached spot determined from the fitting of experimental fluorescence profile, which is often different from *r_n_* due to possible diffusion of fluorophore during the long scanning time of LSCM. By applying Equations (59) and (60) in the definition of FRAP signal recovery, the fluorescence intensity can be calculated from the following correlation:

(61) $$F\left(t\right)=q\int \int \varepsilon I_{r_{n}}\left(x,y\right)\;C\left(x,y,t\right)\;dxdy$$

where *ε* is the attenuation factor of the full laser power in LSCM and *q* is quantum yield. By adopting the same treatment in solving Equation (61) of Axelrod et al. and assuming that nominal bleach radius and actual detection radius may differ $\left(r_{n}\leq r_{e}\right)$, a series of solution of the integral is obtained as follows [71,83]:

(62) $$F\left(t\right)=\overset{\infty }{\underset{m=0}{\sum }}\frac{{\left(-K\right)}^{m}}{m!\left[1+m\left(\frac{2t}{\tau_{D_{e}}}+{\left(\frac{r_{n}}{r_{e}}\right)}^{2}\right)\right]}$$

where $\tau_{D_{e}}=\frac{r_{e}^{2}}{4D_{e}}$ is the diffusion time, and *m* is a series of integers. By setting *m* = 1 in Equation (62) with inclusion of the mobile fraction (*M_f_*), *F*(*t*) will be simplified to the following form:

(63) $$F\left(t\right)=F_{i}\;M_{f}\left\{1-\frac{-K}{1+\gamma^{2}+\frac{2t}{\tau_{D_{e}}}}\right\}+F_{0}\left(1-M_{f}\right)$$

where γ is the ratio for the nominal/effective radius of photobleached spot $\frac{r_{n}}{r_{e}}$; *F_i_* is the steady-state fluorescence intensity before photobleaching; *F_0_* is the fluorescence intensity immediately after photobleaching (*t* = 0). By defining the half-time fluorescence intensity from Equation (63) and $M_{f}=\frac{F_{\infty }-F_{0}}{F_{i}-F_{0}}$, the diffusion coefficient can be determined by experimentally measuring the half-time of fluorescence recovery post bleaching with the use of the following correlation:

(64) $$D=\frac{r_{e}^{2}+r_{n}^{2}}{8\tau_{1/2}}$$

## 3. Emerging FRAP Applications

Using these mathematical models describing the coupled transport/reaction processes for the three types of FRAP, future investigators will be able to explore key frontiers in engineering and cellular physiology.

### 3.1. Physiological Transport

Typical transport processes of biomolecules in physiological systems, e.g., microcirculation, in the presences of steric hindrance, biochemical reactions, and molecular recognitions are critical in delivering drugs to specific sites, such as tumours [97]. The original version of epifluorescence spot FRAP had not been adopted for measuring interstitial diffusive and convective transport of molecules such as antibodies. This was primarily due to the difficulty of distinguishing between different directions of fluid flow in tissues [33]. By using a moderate light source (e.g., Mercury Lamp) instead of a high-power laser beam, Chary and Jain successfully applied FRAP to study diffusion and convection in both normal and neoplastic tissues in vivo (with rabbit ear chamber) without a priori knowledge of the flow direction [33]. Their work showed that the convective flow rate is around 0.6 μm/s in both types of tissues while the diffusion coefficient of fluorescently labelled albumin was increased by 9% in neoplastic tissues (*D* = 6.3 × 10^−7^ cm^2^/s) relative to normal tissues, showing differences in interstitial transport resistance due to disease [13].

Epifluorescence spot FRAP became challenging in thicker samples (with a thickness greater than 50 μm) as the concentration of the fluorophore was no longer linearly correlated with the experimentally detected fluorescence intensity due to light scattering and adsorption [98]. To overcome such challenges, Berk et al. introduced 2D spatial Fourier transform analysis (SFA) on the concentration distribution map obtained with an intensified CCD camera [90]. The group discovered that SFA correctly determined the diffusion coefficient of many biomacromolecules, such as BSA between 4.4 and 600 kDa inside thick agarose gel and tissue mimetics. Moreover, SFA achieved a higher accuracy in the study of diffusive processes in thick light scattering samples (e.g., *D_BSA_* ~ 4.2 × 10^−7^cm^2^/s in 2% agar gel) than direct FRAP analysis [90]. For valid FRAP, the inhomogeneity of biological tissues must be considered. More recently, Sniekers and van Donkelaar have developed a 2D method using Fick’s law to measure localized diffusivity of fluorescein-conjugated BSA in a proximal tibia growth plate. This is a highly inhomogeneous tissue with both high cell and high ECM concentration regions. Their method utilized confocal FRAP [99]. Results indicated a diffusion coefficient for BSA in the ECM of 4.9 × 10^−7^ cm^2^/s, in close agreement with the diffusivity of similar proteins in biological tissues using conventional methods of FRAP analysis, i.e., Equation (8) [13,99].

**Figure 8:**
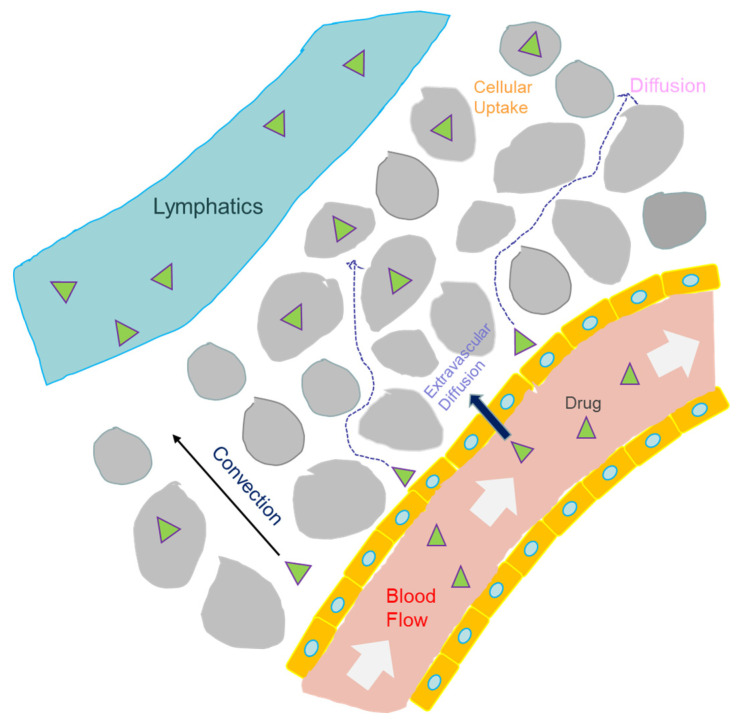
Transport and reaction kinetics involved in drug delivery in circulation, from blood vessel to lymphatics, which are detectable with FRAP techniques.

Physiological transport plays a critical role in the success of drug delivery to specific sites at the interstitial spaces between capillary and lymphatics (Figure 8). The original version of spot epifluorescence FRAP could not measure volumetric flow rates in the microcirculation of typical organs. In 1991, Flamion and co-workers applied a modified FRAP technique with fast fluorescence detection and a revised mathematical model of spot epifluorescence FRAP (using cylindrical geometry) to measure physiological transport in an animal organ [100]. The group applied a short bleaching pulse (20 ms) to measure a volumetric flow rate between 4 and 40 nm/min in the axial direction of perfused kidney tubules from the linear regime of the FRAP curve. Fluorescein sulfonate was used as the tracer [100].

The three major classes of FRAP techniques employ single photon detection, which suffers from the unconfinement of a 3D illumination profile and poor light penetration into biological tissues [101]. By developing a new diffusive-convective transport model for multiphoton (MP)-FRAP, Sullivan and co-workers successfully characterized complex transport processes in thick samples by achieving a greater depth of light penetration [102]. The group demonstrated that a new mathematical model of diffusion-convection in conjunction with MP-FRAP accurately probed the diffusion coefficient of FITC-dextran (9.5 × 10^−8^ cm^2^/s) in one-dimensional convective flow (around 70 μm/s) in tissue capillaries. Their method improved on the diffusion-only model in MP-FRAP [102]. FRAP measurement at submicron scale is difficult with traditional epifluorescence optics and fluorescence detection [103]. Interestingly, Chauhan and co-workers integrated MP-FRAP and SFA-FRAP in one single platform to probe diffusion at different length scales, both the interstitial space and the ECM, within the same field of view [77]. Their unique approach successfully confirmed the role of collagen in hindering diffusion. Such impairment should also apply to drug transport in tissue [77].

Biomolecular transport under conditions such as the altered interstitial fluid pressure in solid tumours is important in the design of drug carriers (Figure 8). For instance, Pluen and co-workers applied epifluorescence spot FRAP to study ECM composition and structure in dorsal skin chamber (DC) or cranial window (CW) implanted tumours in mice [30]. Interestingly, the group demonstrated that increased Type I collagen content in DC tumours led to a reduction of the diffusion coefficient for high MW biomacromolecules (e.g., Immunoglobulin G (IgG)) by at least five-fold in comparison to that in CW tumours. Functional tissues are composed of structured molecular scaffold, a physiological fluid, and cells aligned to produce a highly anisotropic microenvironment [104,105,106]. Travascio and Gu combined SFA-FRAP with multilayer sampling to study the movement of fluorescein in the annulus fibrosus (AF) of intervertebral discs (IVD), a highly anisotropic media consisting of a gel-like matrix surrounded by concentric layers [107]. By measuring the diffusion of fluorescein along three main directions of IVD, the group discovered that the diffusion coefficient of fluorescein in the radical direction of AF was 33% (8.0 × 10^−8^ cm^2^/s) lower than that along an axial or circumferential coordinate, proving diffusional anisotropy. Moreover, the measurement of 3D diffusion tensors simultaneously in highly heterogeneous tissues is challenging in FRAP, as mentioned above [108]. Chen et al. recently developed a new variant of SFA-FRAP by applying a two-photon excitation light sheet and MEMS mirror to generate light-sheet illumination that moves along the sample depth. This leads to the formation of a well-calibrated 3D volume for quantitation of fluorescence recovery after photobleaching [109]. They determined that the average 3D diffusivity of sodium fluorescein in tendons increased from 1.0 to 2.1 × 10^−7^ cm^2^/s during tissue degeneration.

Coupled diffusion and reaction are often involved in cellular signalling, e.g., movement and then binding of growth factors from the fluid phase with specific growth receptors on the plasma membrane to regulate physiological functions [110]. FRAP was applied to mass transport and reaction under both diffusion-limited and reaction-limited binding by using immobilized antigen on Sepharose beads with the antibody in solution [18]. By using reaction-limited binding conditions (illustrated in Equation (31)), the kinetics of binding between B72.3 monoclonal antibodies in the liquid phase and TAG-72 tumour associated antigens (immobilized) or between RCV antibodies in solution and VX2 carcinoma associated antigens (immobilized) were determined [17]. The bulk diffusion coefficient of the B72.3 antibody was 6.2 ± 1.1 × 10^−7^ cm^2^/s independent of concentration between 0.2 and 20 mg/mL. The result agreed well with that reported by Anderson et al. on the diffusion of BSA when the ionic strength was greater than 0.07 M and BSA concentration was less than 29 mg/mL [111]. Importantly, the antibody diffusion coefficient did not differ statistically with the volume fraction of antigen beads in solution. A similar trend of diffusion coefficient versus antibody concentration was seen with the RCV/VX2 system where the diffusion coefficients of RVC-184, RVC-626, and RVC-779 were 4.4 × 10^−7^, 4.8 × 10^−7^, and 4.4 × 10^−7^ cm^2^/s, respectively [95].

In general, epifluorescence spot FRAP was proved to be reliable in obtaining the equilibrium binding constant and the average antigen concentration according to Equations (27) and (28). In the B72.3/TAG72 immobilized bead system, the equilibrium binding constant and the average antigen concentration were 2.5 × 10^7^ M^−1^ and 4.4 × 10^−7^ M, respectively [95]. The validity of the reaction-limited binding model was further confirmed by fluorometry experiments and theoretical simulations of all the physical/chemical processes contributing to FRAP data [18]. The reaction-limited model (low Da) correctly predicted the antibody diffusion coefficient and immobile fraction. In the RVC/VX2 system, RVC-626 was bound more strongly to the VX2 functionalized beads than RVC-184 as shown by the higher equilibrium binding constant of 5.1 × 10^−7^ M^−1^ and average antigen concentration of 5.7 × 10^−7^ M in an 18% volume fraction of antigen beads [18]. By performing a competitive binding assay in which one monoclonal RVC antibody was labelled and incubated with other types of unlabelled RVC antibodies, it was determined that specific antibodies did not cross-complete for the same binding sites on VX2 antigen immobilized on either beads or on antigen presenting cells [17]. The experimental findings strongly supported the expected result that the binding of the antibodies on the antigens is caused by specific immunological interactions.

### 3.2. Interfacial Biophysics

**Figure 9:**
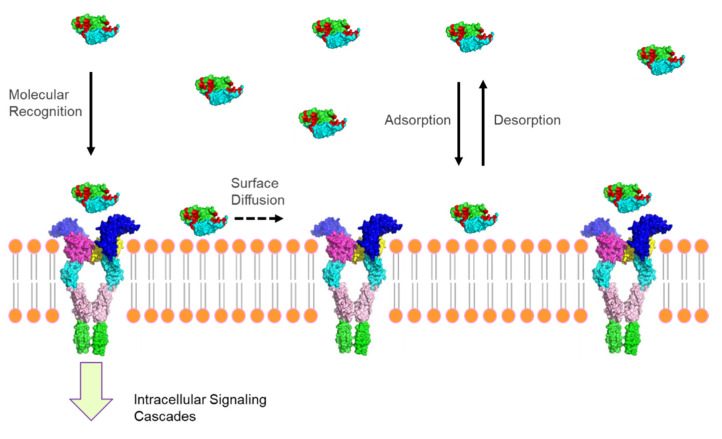
Interfacial phenomena involved in the molecular recognition of biomacromolecule (e.g., ligand) by transmembrane proteins (receptors) on plasma membrane.

Numerous biological processes involve the interaction of biomacromolecules with various surfaces, such as the case of cellular mechanochemical transduction following ligand-receptor binding on a cell membrane. Another example is the use of immobilized enzymes on biocatalytic microparticles [112]. Many molecules either bind specifically with their complement on the cell membrane or adsorb non-specifically on various materials (see Figure 9) [113]. Interestingly, the interfacial association of such molecules is sometimes followed by two-dimensional diffusion on the substrate. This process is well recognized on biological systems, such as cell membranes as well as on artificial biomaterials or biosensors. The conformation, orientation, and function of surface-bound biomacromolecules, such as proteins, can be important to molecular recognitions in biosensors [114].

Several bioanalytical techniques, such as surface plasmon resonance and FT-infrared spectroscopy have been developed to study the kinetics of binding at solid–liquid interfaces under either flow or stagnant conditions [115]. However, the techniques do not allow the measurement of independent parameters involved in molecular recognition or surface diffusion, both of which directly influence the structural and functional properties of molecules at the biointerface [116]. The earliest TIR/FRAP developed in 1981 provides only a rough estimation on the surface diffusion coefficient of surface-bound biomacromolecules due to the difficulty in distinguishing between weakly and strongly associated molecules on the substrates [42]. TIR/fluorescence recovery after pattern photobleaching (FRAPP), a variation of TIR/FRAP, can simultaneously determine surface diffusion coefficients and interfacial association/dissociation rates. It uses a periodic fringe pattern with fringe spacing between 3 to 8 μm. This pattern is created by the interference of two coherent laser beams, resulting in a steady-state sinusoidal distribution of unbleached fluorophores. The dissociation rate constants and surface diffusion coefficients for biomolecules on different surfaces can be determined by analysing the fluorescence intensity after photobleaching such fringe patterns. In 1989, Tilton et al. first developed a new version of TIR/FRAPP to specifically probe the lateral diffusion of strongly adsorbed protein layer on a synthetic polymer surface [117]. The group successfully validated the sole measurement of surface diffusion of strongly adsorbed BSA, an important circulatory protein, on poly(methylmethacrylate) (PMMA) and poly(dimethylsiloxane) (PDMS). They did this by demonstrating proportionality between characteristic fluorescence recovery time and fringe pattern spacing. More interestingly, it was shown that surface diffusion coefficient of BSA on a PDMS surface was 2.1 times higher than that on a PMMA surface (1.2 × 10^−9^ cm^2^/s), due to the difference in hydrophobicity and hydrogen bonding capability [117].

The correlation between surface diffusion, substrate-induced reconfiguration, and intermolecular association of biomacromolecules at solid-liquid interfaces is crucial for improving biocompatibility, bio-specificity, and reliability of biomimetic materials for a wide range of emerging applications, including drug delivery, tissue engineering, and molecular diagnostics [118]. Several groups of biophysicists theoretically predicted that molecular crowding following an increase in surface concentration of molecules would impair the self-diffusion of such molecules due to the formation of impermeable patches [119]. To validate the predictions of biophysical models as mentioned above with experimental study, Tilton and co-workers applied TIR/FRAPP to probe the surface diffusion of BSA irreversibly adsorbed on PMMA against the increase in surface concentration of BSA [45]. The group discovered that the surface diffusion of BSA decreased by one order of magnitude when the fraction of PMMA surface area covered by BSA increased from 0.1 to 0.69 while the mobile fraction of adsorbed BSA remained constant at around 0.4 irrespective to the change of surface concentration. The trend, as mentioned previously, was in good agreement with Saxton’s theoretical model of effective-medium theory and percolation theory, predicting that the excluded volume effect alone significantly hindered the two-dimensional diffusion of adsorbed molecules [119]. Moreover, the physiochemical properties of surfaces are likely to influence the molecular flexibility and surface diffusion of adsorbed proteins through the moderation of intermolecular forces, e.g., hydrogen bonding or electrostatics [120]. More recently, Sonesson and co-workers applied confocal FRAP in conjunction with one-dimensional diffusion analysis to investigate the adsorption and self-diffusion of *Thermomyces lanuginose* (TL)-lipase, a typical protein with detergency properties, against the change of surface hydrophobicity [121]. The group demonstrated that the two-dimensional diffusion coefficient of TL-lipase on the surface was reduced by 70% against the increase in substrate hydrophobicity when contact angle increased from 20° (cleaned glass) to 110° (octadecyltrichlorosilane modified glass), likely due to the exposure of hydrophobic domains and eventual change of molecular conformation of TL-lipase upon adsorption on a highly hydrophobic substrate [121].

A model biological membrane composed of a lipid bilayer has emerged as a popular system for mimicking physiochemical properties of cell membranes in the biophysical characterizations of receptor proteins, ion channels, protein clustering, and membrane fusion [122]. On the other hand, little was known about the correlation between lateral fluidity and 2D surface diffusion of lipid in a model lipid bilayer [123]. Ladha et al. have pioneered the use of epifluorescence spot FRAP to probe the two-dimensional diffusion of N-(7-nitrobenzoyl-2-oxa-1,3-diazol-4-yl)-1,2-dihexadecanoyl-snglycero-3-phosphoethanolamine (NBD-PE) within a planar lipid bilayer fabricated with the Montal–Mueller method above the hole of an observation chamber [124]. The group demonstrated that the surface diffusion coefficient of fluorescently tagged NBD-PE was 1.5 × 10^−7^ cm^2^/s in 1,2-dioleoyl-sn-glycero-3-phosphocholine (DOPC) bilayer while it was reduced by 50% upon the inclusion of around 42% cholesterol in the DOPC bilayer, validating the role of cholesterol in stiffening the biomembrane’s fluid phase [124]. It has been known that the thermotropic transition of the multicomponent lipid bilayer is accompanied by the evolution of microdomain structures detectable by fluorescence microscopy, but less is known about the effect of such thermophysical transformation on the surface diffusion of lipids [125]. Recently, Kure and co-workers developed a new mode of confocal FRAP, known as line mode, for studying the surface diffusion of fluorescently tagged NBD-PC in 2:1 DOPC:1,2-dipalmitoylsn-glycero-3-phosphocholine (DPPC):Cholesterol synthetic biomembrane supported on a glass substrate. They studied the progression of a thermotropic transition within the lipid bilayer [126]. The group demonstrated that surface diffusion coefficient of NBD-PC in 2:1 DOPC:DPPC:Cholesterol biomembrane increased from 2 × 10^−7^ to 3 × 10^−7^ cm^2^/s when the temperature increased from 22 to 38 °C due to the reduction of microdomain size in the lipid bilayer.

The development of a lipopolymer incorporated in a phospholipid-containing synthetic biomembrane was instrumental in creating a longer circulation time and enhanced biocompatibility of novel drug delivery systems. An example includes lipid nanoparticles in the mRNA-based COVID-19 vaccine [127]. However, the hypothesized connection between self-diffusion of lipopolymer, chain density, and biomembrane fluidity has not produced an engineering correlation for the design of more optimized drug delivery vesicles [128]. Zhang and Hill have recently applied confocal FRAP to probe the surface diffusion of 1,2-distearoyl-sn-glycero-3-phosphoethanolamine-N-[poly(ethylene glycol)2000-N’-carboxyfluorescein] (DSPE-PEG2k-CF) within a DOPC lipid bilayer supported on glass substrate under a range of DSPE-PEG2k-CF concentrations [129]. Interestingly, the group demonstrated that the surface diffusion coefficient of DSPE-PEG2k-CF was reduced from 2.5 to 1.0 × 10^−8^ cm^2^/s when the lipopolymer concentration increased from 0.5 to 5 mole % in the DSPE-PEG2k-CF/DOPC lipid bilayer due to hydrodynamic friction and excluded volume thermodynamics encountered by the grafted PEO chain [130].

Weakly adsorbed biomacromolecules, such as proteins, play pivotal roles in the molecular recognition on cell membranes, leading to subsequent mechanochemical transduction into the cell cytoplasm [131]. Thompson and co-workers pioneered the development of TIR/FRAP technique and supporting theories for simultaneous measurements of reaction rate constants and the lateral diffusion coefficient of biomacromolecules as they approach a liquid-solid interfaces (Figure 9) [60,132]. This group successfully formulated tightly coupled theoretical expressions for bulk diffusion, association with surface sites, dissociation from surface sites and surface diffusion of biomacromolecules. They modelled the fluorescence recovery curve from TIR/FRAP experiments under either reaction or diffusion limited conditions. Later, Burghardt and Axelrod built a new TIR/FRAP platform by integrating an external laser beam with an inverted microscope to measure the desorption rate constant and lateral diffusion coefficient of BSA on a plain glass substrate with a wide laser beam (*e*^−2^ half width of 12.5 μm) and a narrow laser beam (*e*^−2^ half width of 2.5 μm), respectively [42]. This team demonstrated that weakly adsorbed BSA simultaneously underwent association/dissociation and surface diffusion at a liquid–solid interface with a rapid dissociation rate and surface diffusion coefficients of 0.26 s^−1^ and 5 × 10^−9^ cm^2^/s, respectively.

The interaction between cell membrane and plasma proteins has been known to affect the apparent enzymatic reaction kinetics of prothrombin conversion involved in blood coagulation [133]. Evanescent-interference illumination used in TIR/FRAAP provides a smaller characteristic length scale (2–10 μm) than a Gaussian-shaped evanescent illumination (50–500 μm). This allows one to measure, very slow, translational motions of proteins on the surface underlying the interfacial association/dissociation process [82,117]. Huang et al. pioneered the application of TIR/FRAAP to probe the adsorption and diffusion of a bovine prothrombin fragment 1 (a model substrate for blood coagulation reaction) on two types of supported planar membranes with distinct thermophysical properties [134]. This group demonstrated that the surface diffusion coefficient and fast desorption rate of bovine prothrombin fragment 1 were 4.8 × 10^−9^ cm^2^/s and 2.7 s^−1^, respectively, on a fluid-like PS/POPC-supported bio-membrane at a low density of weakly adsorbed protein. Not surprisingly, surface diffusion of fragment 1 was retarded at higher surface density and was abolished on solid-like lipid bilayer composed of DPPS/DPPC while fluorescent NBD-PC as a control was found to be mobile on the same biomembrane [134].

Since the beginning of the genomics era, the interfacial behaviour of nucleic acids at a liquid–solid interface has been develop for membrane-based techniques, such as the Southern Blot, and for carrying high throughput assays of DNA/mRNA, e.g., the DNA microarray [135]. Graves and co-workers applied TIR/FRAP and TIR/FRAPP to probe the adsorption/desorption kinetics and surface diffusion of single-stranded DNA oligonucleotide and BSA, respectively, on plain glass and amino-functionalized glass substrates [85]. Our group demonstrated that both BSA and oligonucleotides adsorbed reversibly on plain glass and amine-functionalized glass while surface diffusion of surface-bound oligos was also significant, according to the small fringe pattern. Moreover, fast desorption rates (around 0.2 s^−1^) and surface diffusion coefficients (around 2.1 × 10^−9^ cm^2^/s) of oligonucleotide are of the same order of magnitude as those for BSA on the two surfaces, although the oligonucleotide was 8 times lower in molecular weight [85]. It has been known that the intermolecular forces, such as hydrophobic interactions between biomacromolecules and a functionalized substrate, are critical to many emerging applications ranging from controlled release systems to biomolecular sensors [136]. Our group also applied TIR/FRAPP to probe the interfacial adsorption and diffusion of biomacromolecules on hydrophobic substrates, such as trichlorooctadecyl-silane (ODS) functionalized glass under solutions with a range of dielectric constants [136]. It was shown that fast desorption rates and the surface diffusion coefficient of DNA oligonucleotide on ODS functionalized glass were increased by 65% and 268%, respectively, in comparison to that on positively charged, amino-functionalized glass in accordance with the reduction of density of adsorbed DNA on the hydrophobic surface.

### 3.3. Cellular Dynamics

**Figure 10:**
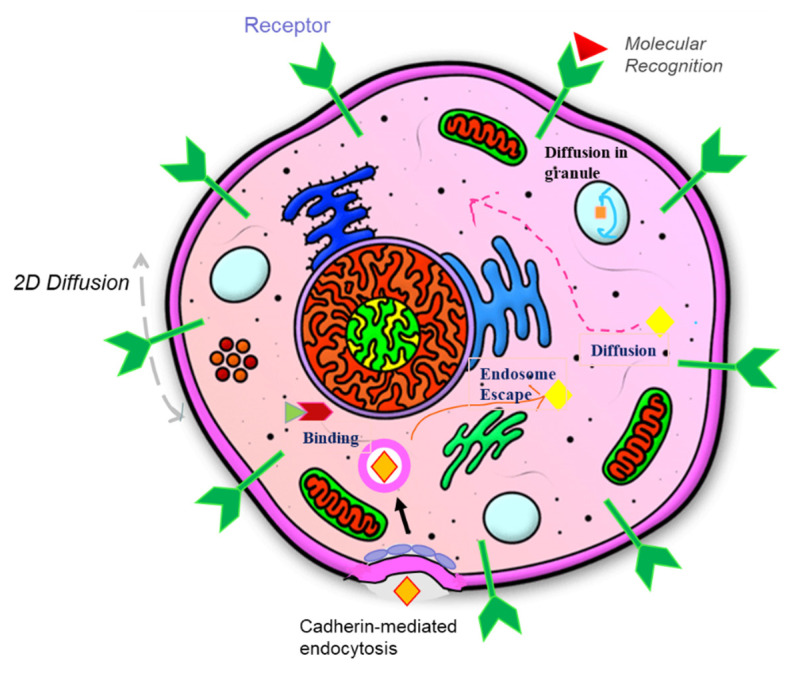
Transport and binding kinetics including ligand-receptor binding, cadherin-mediated endocytosis, diffusion in cytoplasm, diffusion in membrane-bound organelles, escape from endosomes, etc., for maintaining the homeostasis of cellular functions.

With the emergence of GFP technology for functional live-cell imaging, the FRAP technique in conjunction with mathematical modelling has been instrumental for the characterizations of transport-reaction process of molecules within various intracellular compartments, such as membrane-bound organelles and complex bio-interfaces [87]. Figure 10 shows a series of biophysical processes, including ligand-receptor binding, cadherin-mediated endocytosis, diffusion in cytoplasm, diffusion in membrane-bound organelles, escape from endosomes, etc., pertaining to biochemical signal transduction in live cells at homeostasis or under pathophysiological responses. Among various membrane-bound organelles, endoplasm reticulum (ER), which is filled with very high concentration of enzymes at high ionic strength, likely exhibits impaired molecular diffusion. This regulates the protein folding pathway and subsequent protein translocations to the cytoplasm or Golgi apparatus [137]. Dayel et al. applied ER-targeted GFP to introduce fluorescently tagged molecules into the ER lumen of live CHO cells to probe diffusive transport in a highly constrained environment. They used epifluorescence spot FRAP [138] in their study. The group demonstrated that the diffusion coefficient of GFP in the ER lumen (7.5 × 10^−8^ cm^2^/s) was reduced by 13-fold and 4-fold compared to that in water and cytoplasm, respectively, independent of the biomolecular binding of GFP to the ER structure. The trend of hindered diffusion of biomacromolecules in the ER lumen was likely caused by the collision with molecular obstacles, increase of medium viscosity, and solute concentration, showing a strong dependency on the physiochemical properties of the ER compartment [138].

Different membrane-bound organelles consist of unique aqueous compartments, which are filled with different amounts of molecules, such as enzymes and ions. This would be expected to lead to various degrees of hindrance to biomolecular diffusion [139]. Verkman and coworkers combined GFP expression technology and epifluorescence spot FRAP to probe the translational diffusion of biomacromolecules in several types of mitochondrial matrices in live cells, including CHO, HepG2, and LLC-PK1 [140]. By applying a 1D diffusion model in FRAP analysis, the group demonstrated that the diffusion coefficient of GFP inside mitochondria (2.5 × 10^−7^ cm^2^/s) was around 4-fold lower than that in water, while negligible diffusion was detected from GFP fused with fatty acid β-oxidation multienzyme complex. Interestingly, the diffusion coefficient of GFP was unexpectedly low, consistent with the fact that mitochondria have the highest concentration of enzymes or proteins compared to other organelles. The localization of proteins at the periphery of inner mitochondria membranes rather than just being present in solution is also likely to be a factor [140].

The highly intricate interplay between diffusion and binding of nuclear proteins presents significant challenges in determining the binding kinetics involved in gene expression [141]. To determine the effect of nuclear protein binding on molecular transport, Sprague et al. expressed either GFP or GFP-tagged glucocorticoid receptor (GR) in the nucleus of Mouse adenocarcinoma cell line 3617. FRAP measurements followed by the analysis were used along with full reaction-diffusion equations and other simplified models [142]. The group discovered that the confocal-FRAP data of GFP-GR (GR being a protein which binds to the nuclear matrix) was not fitted well by either pure diffusion- or reaction-dominant model, while it was well-modelled by a full set of reaction-diffusion equations. At the same time, their highly quantitative approach successfully disentangled the contributions of diffusion, binding, ATP depletion, and the number of binding states from the trend of fluorescence recovery in FRAP measurement of the cell nucleus [142].

Once mRNA has been synthesized by RNA polymerase II inside the nucleus of eukaryotic cells, it goes through a cascade of diffusion and binding in nucleoplasm, leading to the necessary modification of mRNA for the eventual export to cytoplasm through the nuclear pore complex (NPC) [143]. To probe the coupled diffusion-reaction of mRNA in vivo with FRAP, a fluorescent probe with low molecular weight and mRNA binding affinity, such as GFP-Poly(A) Binding Protein Nuclear 1 (PABPN1), is required to label the otherwise optically inactive biomacromolecules within nucleoplasm. This leads to the simultaneous presence of two fluorescently labelled species: (GFP- PABPN1 and mRNA/GFP-PABPN1 complex) [144]. Braga et al. extended the reaction-diffusion model to account for the coexistence of two fluorescently labelled species to estimate an accurate diffusion coefficient of high molecular weight species from confocal-FRAP data [145]. This group first applied a simple diffusion model in FRAP data analysis to estimate that the diffusion coefficient of mRNA (complexed with GFP- PABPN1 tag) in the nucleus of Hela cell to be 6 × 10^−9^ cm^2^/s. In contrast, the diffusion coefficient of the mRNA-bound complex was significantly lower (4 × 10^−10^ cm^2^/s) when the data was fitted with the reaction-diffusion model. The result strongly indicated that over-estimation of the mRNA diffusion coefficient was caused by the omission of the binding affinity between mRNA and molecular fluorescent probes, such as GFP- PABPN1 in FRAP data analysis. This omission ignored the reversible reaction between GFP- PABPN1 and poly(A)mRNA coupled to the molecular diffusions of individual species [145].

Compared to the nucleus and ER, which are mainly engaged in the synthesis of biomacromolecules, the Golgi apparatus (GA) operates as the logistic centre of protein transport between cytoplasm and the external microenvironment through major organelles, such as the endosome and ER, as well as forming secretary vesicles [146]. The molecular assembly of coat protein I (COPI) into vesicles regulated by the ADP ribosylation factor 1 (ARF1) and its GTPase-activating protein (ARFGAP1) is a critical step for the recycling of newly synthesized proteins from GA back to the ER [147]. Elsner et al. pioneered the applications of fluorescence correlation spectroscopy and confocal-FRAP to investigate the diffusional characteristics and binding kinetics of COPI coatomer or ARF1 or ARFGAP1 to GA membrane in either HeLa or CHO cells [56]. This group demonstrated that the diffusion coefficient of either ARFGAP1 or ARF1 (1.5 × 10^−7^ cm^2^/s) was around 10 times higher than that of the COPI coatomer, which was assembled from the monomeric GFP-COPI into the larger coatomer. Confocal-FRAP measurement showed that the binding rate of GFP-COPI to GA membrane was two times slower than that of ARF1 or ARFGAP1, indicating the diffusion-limited binding kinetics of larger COPI coatomer [56]. More specifically, the transport of fully processed proteins from GA to the extracellular microenvironment was mediated by the budding of secretory granules (which encapsulate granule lumen proteins) from the trans-Golgi network followed by the fusion of mature secretory granules with the inner leaflet of the cell membrane [148].

Weiss et al. pioneered the application of TIR/FRAP in conjunction with GFP technology to probe the real-time diffusion of two types of resident proteins, including tissue plasminogen activator (tPA) and neuropeptide Y (NPY), in secretary granules of living chromaffin cells. Their work is relevant to the post-fusion release kinetics of granule lumen proteins to the extracellular microenvironment [149]. The group demonstrated that the diffusion coefficient of tPA (2 × 10^−10^ cm^2^/s) inside the secretory granules was significantly lower than that of NPY, approaching a 3000-fold reduction compared to a protein of similar size in aqueous solution. The trend of slow tPA diffusion strongly indicated that the protein interacted with the fusion pore of secretory granules, leading to pore stabilization and delaying pore expansion. This limits its own mobility and post-fusion release efficiency [150]. In addition to the roles that the plasma membrane plays in protein secretion, it hosts integral membrane proteins ranging from adhesion receptors to ion channels, G-proteins, enzymes, intracellular junctions, etc. [151]. For instance, Transient Receptor Potential (TRP) channels are a critical part of the membrane, driving sensory and maintenance functions in mammals. They underlie as of yet relatively unknown mechanisms of intracellular trafficking to guide their expressions on plasma membrane [152]. Ghosh et al. applied TIR/FRAP in conjunction with GFP fusion technology to probe the dynamics of membrane incorporation and lateral diffusion for two types of TRP channels, including GFP-TRPM4 and GFP-TRPV2 on plasma membranes in HEK293 cells [153]. The group demonstrated that there were two distinct and unexpected mechanisms of TRP channel turnover by monitoring the kinetics of repopulation of the channel proteins at the plasma membrane/glass coverslip interface. Their results indicated that the perimembrane dynamics of GFP- TRPV2 were controlled by lateral diffusion (with diffusion coefficient of 3 × 10^−9^ cm^2^/s) within the plasma membrane, while fluorescence recovery of GFP-TRPM4 was mediated by fusion of transport vesicles [153].

## 4. Conclusions

The recent advancement of omics technology has opened new doors for high throughput mapping of structure–property–function relationships of many proteins encoded by the human genome. In conjunction with GFP fusion technology, omics technology allows researchers to express fluorescently tagged protein reporters with high spatial-temporal resolution in any cellular organelle for biochemical and biophysical studies. In particular, FRAP, which hinges on the measurement of intrinsic dynamics of molecules after deactivation of photoactive reporters is applicable to experiments in transport phenomena, biophysics and bio-interfacial phenomena. In this work, the authors have given a holistic review on emerging applications of three major modes of FRAP. The mathematical theory for each mode of FRAP, including rate processes, such as convection, diffusion, and chemical reaction, was systematically described. Then, notable recent advances were reviewed. The authors hope that this exposition of the capabilities of FRAP will encourage its application in emerging areas of cellular physiology.

## Figures and Tables

**Table 1:** Comparisons of various types of experimental methods for the measurement of biomolecular kinetics and/or transport processes.

Types of Biophysical Techniques	Measurable Processes	Limitations	References
Fluorescence Recovery After Photobleaching	Convection	Require sophisticated models;	[13]
	Diffusion	require high-powered lasers;	[45]
	Reaction/Binding	only valid for large ROI *	[60]
Fluorescence Correlation Spectroscopy	Diffusion	Lack of interpreting models;	[56]
	Reaction/Binding Concentration	difficult to apply in live cells; require high S/N * ratio	[61] [62]
Single Particle Tracking	Diffusion	Only for dilute species;	[22]
	Viscosity	measure lower mobility;	[55]
	Molecular Binding	requires feedback tracking	[63]
Surface Plasmon Resonance Sensor	Reaction/Binding	Require gold substrate; lack of	[52]
	Mass transfer	interpreting models; noise from used optoelectronics	[64] [65]
Stochastic Optical Reconstruction	Diffusion	High costs; require photo-	[66]
	Molecular Binding	switchable dyes as labels; require experts in operation	[67] [68]
	Molecular Interaction	Complicated mathematical	[57]
FCCS ^#^	Composition/Fraction Large Complex	modeling; supplicated instrumentation; high cost	[69] [70]

* Signal to noise: S/N; Region of Interest: ROI. ^#^ Fluorescence Cross-Correlation Spectroscopy: FCCS.

## Data Availability

Not applicable.
